# A Prospective Randomized, Double-Blind, Multi-Center, Phase III Clinical Trial Evaluating the Efficacy and Safety of Olmesartan/Amlodipine plus Rosuvastatin Combination Treatment in Patients with Concomitant Hypertension and Dyslipidemia: A LEISURE Study

**DOI:** 10.3390/jcm11020350

**Published:** 2022-01-11

**Authors:** Sang-Ho Jo, Seok Min Kang, Byung Su Yoo, Young Soo Lee, Ho Joong Youn, Kyungwan Min, Jae Myung Yu, Hyun Ju Yoon, Woo Shik Kim, Gee Hee Kim, Jae Hyoung Park, Seok Yeon Kim, Cheol Ho Kim

**Affiliations:** 1 Department of Internal Medicine, Division of Cardiology, Hallym University Sacred Heart Hospital, Anyang 14068, Korea; sophi5neo@gmail.com; 2 Department of Internal Medicine, Yonsei University College of Medicine, Seoul 03722, Korea; SMKANG@yuhs.ac; 3 Department of Internal Medicine, Division of Cardiology, Wonju College of Medicine, Yonsei University, Wonju 26426, Korea; yubs@yonsei.ac.kr; 4 Department of Internal Medicine, Division of Cardiology, Daegu Catholic University Medical Center, Daegu 42472, Korea; mdleeys@cu.ac.kr; 5 Department of Internal Medicine, Division of Cardiology, Seoul St. Mary’s Hospital, The Catholic University of Korea, Seoul 06591, Korea; hjy@catholic.ac.kr; 6 Nowon Eulji Medical Center, Department of Internal Medicine, Division of Endocrinology, Eulji University, Seoul 01830, Korea; minyungwa@gmail.com; 7 Department of Internal Medicine, Division of Endocrinology, Hallym University Kangnam Sacred Heart Hospital, Seoul 07441, Korea; jaemyungyu@hotmail.com; 8 Department of Internal Medicine, Division of Cardiology, Chonnam National University Hospital, Gwangju KS018, Korea; ann426@hanmail.net; 9 Department of Cardiology, Kyunghee Medical Center, Seoul 02447, Korea; wskim1125@khu.ac.kr; 10 Department of Internal Medicine, Division of Cardiology, St. Vincent’s Hospital, College of Medicine, The Catholic University of Korea, Seoul 06591, Korea; jiheekim@catolic.ac.kr; 11 Department of Internal Medicine, Division of Cardiology, Korea University Anam Hospital, Seoul 02841, Korea; jhpark3992@naver.com; 12 Department of Internal Medicine, Division of Cardiology, Seoul Medical Center, Seoul 02053, Korea; ks7688@hanmail.net; 13 Department of Internal Medicine, Division of Cardiology, Seoul National University Bundang Hospital, Seongnam 13620, Korea

**Keywords:** olmesartan, amlodipine, rosuvastatin, single pill combination, phase III clinical trial

## Abstract

Background: This study was a multicenter, randomized, double-blinded, placebo-controlled phase III clinical trial to investigate the efficacy and safety of an olmesartan/amlodipine single pill plus rosuvastatin combination treatment for patients with concomitant hypertension and dyslipidemia. Methods: Patients with both hypertension and dyslipidemia aged 20–80 were enrolled from 36 tertiary hospitals in Korea from January 2017 to April 2018. Patients were randomized to three groups in a 1:1:0.5 ratio, olmesartan/amlodipine single pill plus rosuvastatin (olme/amlo/rosu) or olmesartan plus rosuvastatin (olme/rosu) or olmesartan/amlodipine single pill (olme/amlo) combination. The primary endpoints were change of sitting systolic blood pressure (sitSBP) from baseline in the olme/amlo/rosu vs. olme/rosu groups and the percentage change of low-density lipoprotein cholesterol (LDL-C) from baseline in the olme/amlo/rosu vs. olme/amlo groups after 8 weeks of treatment. Results: A total of 265 patients were randomized, 106 to olme/amlo/rosu, 106 to olme/rosu and 53 to olme/amlo groups. Baseline characteristics among the three groups did not differ. The mean sitSBP change was significantly larger in the olme/amlo/rosu group with −24.30 ± 12.62 mmHg (from 153.58 ± 10.90 to 129.28 ± 13.58) as compared to the olme/rosu group, −9.72 ± 16.27 mmHg (from 153.71 ± 11.10 to 144.00 ± 18.44 mmHg). The difference in change of sitSBP between the two groups was −14.62± 1.98 mmHg with significance (95% CI −18.51 to −10.73, *p* < 0.0001). The mean LDL-C reduced significantly in the olme/amlo/rosu group, −52.31 ± 16.63% (from 154.52 ± 30.84 to 72.72 ± 26.08 mg/dL) as compared to the olme/amlo group with no change, −2.98 ± 16.16% (from 160.42 ± 32.05 to 153.81 ± 31.57 mg/dL). Significant difference in change was found in LDL-C between the two groups with −50.10 ± 2.73% (95% CI −55.49 to −44.71, *p* < 0.0001). Total adverse drug reaction rates were 10.48%, 5.66% and 3.7% in the olme/amlo/rosu, olme/rosu and olme/amlo groups, respectively with no statistical significance among the three groups. Serious adverse drug reactions did not occur. Conclusions: Olmesartan/amlodipine single pill plus rosuvastatin combination treatment for patients with both hypertension and dyslipidemia is effective and safe as compared to either olmesartan plus rosuvastatin or olmesartan plus amlodipine treatment.

## 1. Introduction

Single pill combination (SPC) of two or more antihypertensive drugs has shown promising results in improving drug compliance, lowering blood pressure and potentially providing better clinical outcomes [1,2,3,4].

There have been many clinical trials to evaluate the efficacy and safety of the SPC of 2–3 classes of antihypertensive or dyslipidemia drug [5,6,7]. Moreover, SPCs with both antihypertensive and anti-dyslipidemia drugs have been developed and tested [4,8,9,10,11]. Most of these studies showed promising efficacy and safety data as compared to monotherapy or equivalent doses of separate pill combinations. Reflecting these results, recent guidelines regarding hypertension management specifically indicated the use of single-pill combinations for the simple purpose of improving drug adherence [12]. The concept of single pill, or fixed dose combination, or poly-pill has broadened its scope beyond hypertension or dyslipidemia treatment. A recent clinical trial assessing the separate small dose combination of four classes of cardiovascular drugs suggests promising use of the poly-pill in reducing cardiovascular disease [10,13]. The performance of this kind of clinical trial suggests the popularity and promising future directions for the poly=pill or SPC in treating hypertension and cardiovascular disease. Patients with hypertension had high probability of dyslipidemia and vice versa. Therefore, concomitant prescription of antihypertensive and anti-dyslipidemia drugs is not rare and this will increase in the future as the elderly and co-morbid population grows. Accordingly, the SPC of different kinds of antihypertensive and anti-dyslipidemia drugs is needed in the context of compliance improvement and for better clinical outcome. Moreover, reflecting the recommendation of recent hypertension treatment guidelines regarding the initial two-drug combination for blood pressure management, even in stage I hypertensive patients, a three-drug combination with two antihypertensive drugs and one anti-dyslipidemia drug for patients with combined risk of hypertension and dyslipidemia is a reasonable strategy and needs to be tested [12]. We tested a well-known drugs combination, olmesartan and amlodipine as antihypertensive medication, and rosuvastatin as an anti-dyslipidemia drug. Olmesartan showed signs of harm by a greater occurrence rate of fatal cardiovascular events in a large scale prospective clinical, even if it showed promising results in reducing microalbuminuria [14]. However, other clinical trials showed safety and benefits in preventing cardiovascular events [15,16]. Furthermore, olmesartan is one of the most widely used antihypertensive drugs in Korea and globally [17].

We performed a prospective multi-center randomized double blinded placebo-controlled phase III clinical trial to assess an SPC drug composed of olmesartan/amlodipine plus a separate dose of rosuvastatin to examine its efficacy and safety in controlling blood pressure and serum lipid level. The final study purpose was to develop a triple SPC with these three drugs.

## 2. Methods

### 2.1. Purpose

This multicenter double blind phase III clinical trial was performed from January 2017 to April 2018 in 36 tertiary hospitals in Korea. The study purpose was to test the blood pressure (BP) lowering and low-density lipoprotein cholesterol (LDL-C) reducing effect of olmesartan/amlodipine plus a separate dose of rosuvastatin as compared to olmesartan plus rosuvastatin or olmesartan plus amlodipine.

The trial protocol was approved by the institutional review board at Seoul National University Bundang Hospital (IRB No. B-1610-368-002) and Hallym University Sacred Heart Hospital (IRB No. 2016-S072). All patients provided written informed consent at the time of enrolment and randomization. This study was performed under the standards specified in the International Council for Harmonization Guidelines for Good Clinical Practice and the principles of the Declaration of Helsinki. This trial was registered at ClinicalTrials.gov, with Identifier NCT03009487.

### 2.2. Patients

Patients aged 20–80 who had concomitant hypertension and dyslipidemia participated in this study. The patients had to have been prescribed both anti-hypertensive and anti-dyslipidemic drugs or meet the diagnosis criteria for hypertension and dyslipidemia (Supplementary Tables S1 and S2).

Exclusion criteria were (1) mean sitting systolic BP (sitSBP) difference of 20 mmHg or over among three readings or sitDBP 10 mmHg or over, (2) mean sitting diastolic BP (sitDBP) ≥ 110 mmHg in three readings at randomization visit (visit 2), (3) compliance to olmesartan 40 mg was under 70% or over 130%, (4) those with symptomatic orthostatic hypotension, (5) likely to have secondary hypertension, (6) severe heart failure with NYHA class III-IV symptom, severe aortic or mitral stenosis, those with obstructive hypertrophic cardiomyopathy or severe coronary obstructive disease, (7) critical arrhythmia, (8) those with stroke or transient ischemic attack, acute coronary syndrome, peripheral arterial disease or coronary revascularization within 6 months, (9) uncontrolled diabetes (HbA1c ≥ 9.0% or fasting blood glucose ≥ 160 mg/dL), (10) uncontrolled thyroid abnormalities, (11) chronic kidney disease (serum creatinine ≥ 2 mg/dL or renal replacement therapy or hepatic failure patients and with AST or ALT over 2 times of upper normal limit, (12) Crohn’s disease, active hemorrhagic ulcer and acute and chronic pancreatitis, (13) chronic inflammation status requiring anti-inflammatory drugs, (14) myopathy or history of rhabdomyolysis, (15) critical hyperuricemia (uric acid > 10 mg/dL) or hyperkalemia (K > 5.5 mmol/L), (16) clinically meaningful hyponatremia (Na < 130 mmol/L) or volume depletion status, (17) malignancy within 5 years, (18) alcohol or drug abuser within 1 year, (19) pregnancy or breast feeding.

### 2.3. Study Drugs

The study drug was olmesartan 40 mg/amlodipine 10 mg (SPC) plus rosuvastatin 20 mg, olme/rosu group drug was olmesartan 40 mg plus rosuvastatin 20 mg (separate drug), and olme/amlo group drug was olmesartan 40 mg/amlodipine 10 mg (single pill combination). Study drug group received olmesartan 40 mg/amlodipine 10 mg (2 drug single-pill) plus rosuvastatin 20 mg plus placebo of olmesartan 40 mg. Olme/rosu group received olmesartan 40 mg plus rosuvastatin 20 mg plus placebo of olmesartan 40 mg/amlodipine 10 mg and olme/amlo group patients received olmesartan 40 mg/amlodipine 10 mg plus placebo of olmesartan 40 mg plus placebo of rosuvastatin 20 mg.

### 2.4. Study Procedures

**Figure 1:**
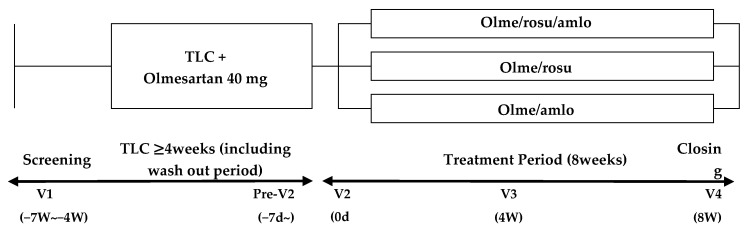
Study scheme. TLC, therapeutic life style change; olme, olmesartan; rosu, rosuvastatin; amlo, amlodipine; v, visit.

After screening for eligibility (visit 1) and before randomization (visit 2), if subjects already took these medications, they stopped both antihypertensive and anti-dyslipidemia medications including fenofibrate, and took only olmesartan at 40 mg per day combined with therapeutic life style changes after patients agreed and provided informed consent at study entrance for 6 weeks of washout period (Figure 1).

Their sitSBP ought to have been within 140 to 180 mmHg at randomization time point (visit 2) and their lipid profile ought to have met the criteria (visit 2) (Figure 1, Supplementary Tables S1 and S2). The BP was measured at the arm, with higher BP taken. After checking the BP and lipid profile at visit 2, patients were randomly assigned to three groups consecutively, study drug group (olme/amlo/rosu), olme/rosu and olme/amlo groups, with 1:1:0.5 ratio and with PROC plan in SAS system V9.3. by an independent statistician who do not know the study performance. We allocated half the number of patients to the/amlo group as compared to the other groups because of an ethical issue; those in the olme/amlo group did not receive anti-dyslipidemia drugs.

**Figure 2:**
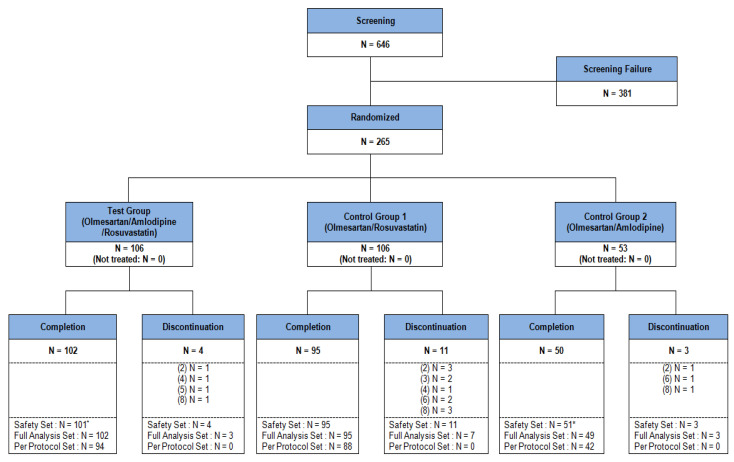
Study patients flow. * = 1 person was classified into olme/amlo group instead of olme/amlo/rosu group in safety analysis due to study investigational product was falsely distributed.

After being randomized to each group, patient received investigational drugs for 8 weeks and were followed up for safety and efficacy at 4 and 8 weeks of treatment (Figure 2).

Drug compliance was estimated by checking the remaining drugs at visit 2 and calculating real intake dose/planned dose.

#### 2.4.1. Sample Size Estimation and Statistical Analysis

For sample size estimation in the aspect of sitSBP change, the sitSBP change was −21.53 mmHg in amlo group and −13.30 in non-amlo group, with maximal standard deviation of 16.58 mmHg.

Thus, null hypothesis was *H*_0_ = *µ_t_* − *µ_c_* ≥ 0

$\mu_{t}$: Mean sitSBP change in Olmesartan/Amlodipine/Rosuvastatin group.

$\mu_{c}$: Mean sitSBP change in Olmesartan/Rosuvastatin group.

$n_{}=\frac{2{\left({Ζ}_{\alpha }+Z_{\beta }\right)}^{2}\sigma^{2}}{{\left(\mu_{t}-\mu_{c}\right)}^{2}}=\frac{2\times {\left(1.96+1.282\right)}^{2}\times 16.58^{2}}{{\left(-8.23\right)}^{2}}=85.32\approx 86$

Thus, 86 patients in each group were required to allow for 2.5% alpha and 10% beta error.

For samples size estimation in the aspect of LDL-C change, we referred to relevant references and found minimal −48.0% LDL-C difference between rosuvastatin and no-rosuvastatin groups. We assumed maximal standard deviation as 11.1% and we hypothesized as follows;

Thus, null hypothesis was *H*_0_ = *µ_t_* − *µ_c_* ≥ 0

$\mu_{t}$: LDL-C change in Olmesartan/Amlodipine/Rosuvastatin group

$\mu_{c}$: LDL-C change in Olmesartan/Amlodipine group

$n_{}=\frac{2{\left({Ζ}_{\alpha }+Z_{\beta }\right)}^{2}\sigma^{2}}{{\left(\mu_{t}-\mu_{c}\right)}^{2}}=\frac{2\times {\left(1.96+1.282\right)}^{2}\times {\left(0.111\right)}^{2}}{{\left(-0.48\right)}^{2}}=1.12\approx 2$

Thus, two patients in each group were required to allow for 2.5% alpha and 10% beta error.

Combining the above two calculations, final study population was 86 in each group. Considering 15% lost to follow-up, 102 patients in the olme/amlo/rosu and olme/rosu groups and 51 in the olme/amlo group were required with 1:1:0.5 ratio.

The efficacy was evaluated mainly with a full analysis set (FAS) along with a Per-protocol Set (PPS) and safety was tested with a safety set (SS). In dealing with the missing values for the efficacy analysis, we used Last Observation Carried Forward (LOCF) methods for adjustment, and for safety analysis we used original data, with missing for without LOCF. Categorical variables were presented with numbers and percentages and compared using the Pearson’s chi-square test or Fisher’s exact test. Continuous variables with normal distribution were presented as the mean ± standard deviation and compared using the paired sample *t*-test, or one sample *t*-test or Wilcoxon singed rank test for intra-group comparison. To test the inter-group differences for continuous variables, ANCOVA test was performed. *p* value of <0.05 was considered to be significant. Statistical analysis was carried out using SAS version 9.4.

#### 2.4.2. Endpoints

Primary endpoints were sitSBP change from baseline at 8 week treatment in olme/amlo/rosu vs. olme/rosu group and percentage change of LDL-C from baseline in olme/amlo/rosu vs. olme/amlo group. The differences from baseline between two groups in sitSBP and LDL-C were also compared (study drug group vs. each of two comparator groups).

Secondary endpoint was (1) sitSBP change from baseline in olme/amlo/rosu and olme/amlo, (2) LDL-C change from baseline in olme/amlo/rosu and olme/rosu, (3) sitDBP change from baseline in olme/amlo/rosu, olme/rosu and olme/amlo, (4) target BP and LDL-C attainment rate in olme/amlo/rosu vs. olme/rosu vs. olme/amlo.

(5) Total cholesterol, triglyceride (TG), HDL-C, APO-A1 and AP-B change.

## 3. Results

A total of 646 patients participated in the study from 29 tertiary hospitals in Korea. After 381 patients dropped out in screening, 265 patients were randomized. FAS (patients taking drug at least once and having efficacy evaluation at least once) included 259 (olme/amlo/rosu group 105, olme/rosu group 102, olme/amlo group 52), and per-protocol set (completing the study) included 224 patients (olme/amlo/rosu group 94, olme/rosu group 88, olm/amlo group 42) and safety set (patients taking drug at least once and followed-up at least once) included 265 patients (olme/amlo/rosu group 105, olme/rosu group 106, olm/amlo group 54) (Table 1, Figure 2, Supplementary Table S3). The overall drug adherence rate was 97.42 ± 4.73%. In the individual group, the rates were 97.01 ± 5.29% in olme/amlo/rosu, 98.24 ± 2.75% in olme/rosu and 96.65 ± 6.24% in olme/amlo group, with no statistical difference among the three groups (*p* = 0.9706).

### 3.1. Efficacy Outcomes

#### 3.1.1. Primary Endpoint 

The primary outcome was comparison of sitSBP change after 8 weeks of treatment between olme/amlo/rosu (*n* = 105) vs. olme/rosu groups (*n* = 102) and comparison of LDL-C change between olme/amlo/rosu vs. olme/ amlo groups (FAS analysis). The mean sitSBP change was significantly larger in olme/amlo/rosu group with −24.30 ± 12.62mmHg (from 153.58 ± 10.90 to 129.28 ± 13.58) as compared to olme/rosu group, −9.72 ± 16.27 mmHg (from 153.71 ± 11.10 to 144.00 ± 18.44 mmHg) (Table 2 and Figure 2). The change of sitSBP from baseline at 8 weeks was more pronounced in olme/amlo/rosu group as compared to olme/rosu group, and the difference of change between 2 groups was −14.62 ± 1.98 mmHg with significance (95% CI −18.51 to −10.73, *p* < 0.0001). (Table 2 and Figure 2).

**Figure 3:**
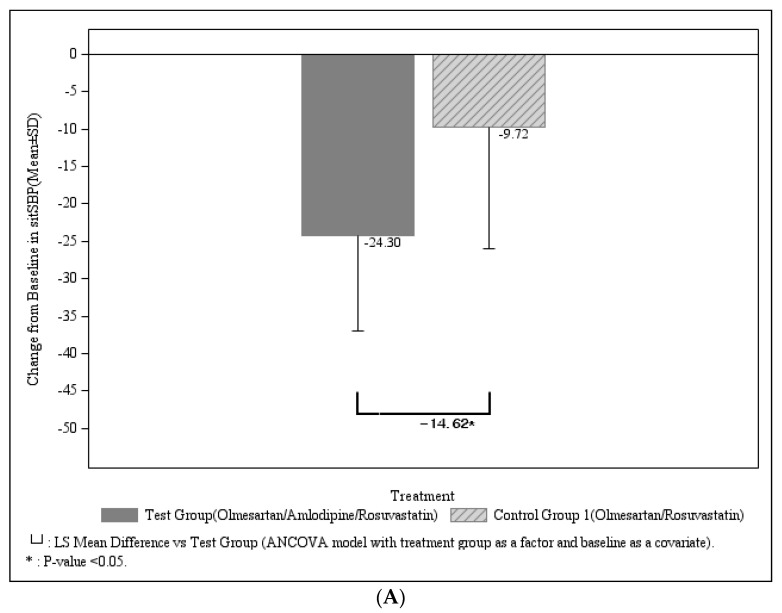

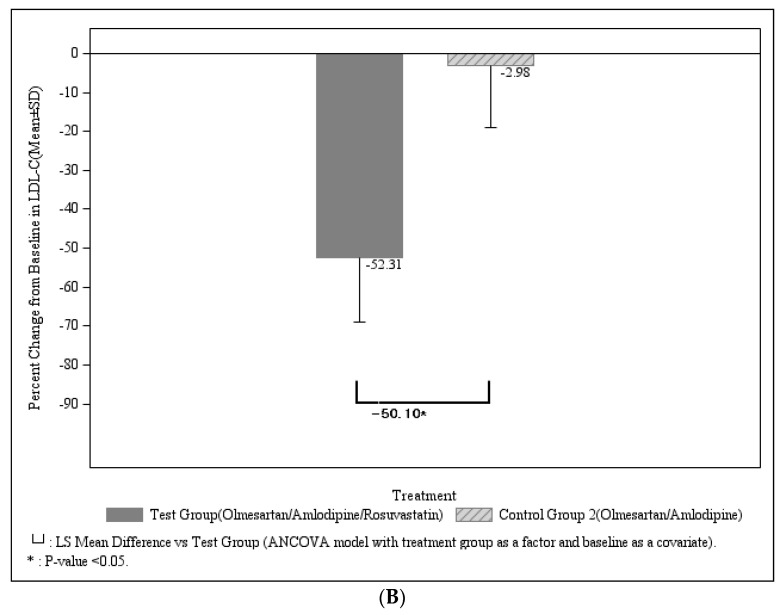
(**A**) Change of sitSBP in olme/amlo/rosu vs. olme/rosu after 8 week treatment (**B**) Change of LDL-C in olme/amlo/rosu vs. olme/amlo after 8 week treatment.

The mean LDL-C percentage reduction was significantly more in the olme/amlo/rosu group, −52.41 ± 16.63% (from 154.52 ± 30.84 to 72.72 ± 26.08 mg/dL) compared to that of the olme/amlo group, −2.98 ± 16.16% (from 160.42 ± 32.05 to 153.81 ± 31.57 mg/dL) with *p* < 0.0001. More LDL-C reduction was found in the olme/amlo/rosu group compared to the olme/amlo group, with a significant difference of change between the two groups with −50.10 ± 2.73% (95% CI −55.49 to −44.71, *p* < 0.0001) (Table 3 and Figure 3).

By per-protocol set analysis, sitSBP significantly reduced by −24.60 ± 12.0 mmHg in the olme/amlo/rosu group (*n* = 94) and −9.93 ± 14.90 mmHg in the olme/rosu groups (*n* = 88) at 8 weeks with statistical significance (both *p* < 0.0001). Inter-group difference in sitSBP change from baseline was −14.57 ±1.95 mmHg with significance (*p* < 0.0001). With per-protocol set analysis by ANCOVA, LDL-C was significantly reduced by −52.92 ± 15.23% in the olme/amlo/rosu group (*p* < 0.0001) and but not reduced in the olme/amlo group, −3.10 ± 17.27% (*p* = 0.2513). The change of LDL-C from baseline is significantly higher in the olme/amlo/rosu group; inter-group difference of LDL-C change was −50.06 ± 2.9%.

#### 3.1.2. Secondary Endpoints

The sitSBP change from baseline between olme/amlo/rosu vs. olme/amlo were −24.30 ± 12.62 mmHg and −22.89 ± 11.74 mmHg, respectively, with no difference (LS mean difference (SE), −0.63 ± 2.02 mmHg, *p* = 0.7555) despite that intra-group BP reduction in each group was significant (both *p* < 0.0001). The LDL-C change in olme/amlo/rosu was −52.31 ± 16.63% and −51.38 ± 17.46% in olme/rosu group at 8-week with statistic significance in each intra-group, but inter-group difference of change between 2 groups was −1.34 ± 2.33% with no difference (*p* = 0.5667).

The change of sitDBP in olme/amlo/rosu vs. olme/rosu vs. olme/amlo at 4- and 8-week were −11.83 ± 7.52 mmHg, −4.23 ± 8.52 mmHg, −10.21 ± 7.50 mmHg and −12.06 ± 7.81 mmHg, −4.72 ± 9.01 mmHg, −12.46 ± 7.01 mmHg, respectively, with statistical significance in each group (all *p* < 0.0001 as compared to baseline in each group at 4 and 8 weeks). The change of sitDBP in olme/amlo/rosu at 4 and 8 weeks was significantly greater as compared to that of olme/rosu with group difference of −8.53 ± 1.07 mmHg (LS mean difference (SE), *p* < 0.0001) at 4 weeks and −8.33 ± 1.11mmHg (LS mean difference [SE], *p* < 0.0001) at 8 weeks, but the difference of sitDBP change from baseline at 4 and 8 weeks between olme/amlo/rosu vs. olme/amlo were not different with LS mean difference (SE), −1.51 ± 1.12 mmHg, (*p* = 0.1806) and 0.57 ± 1.14 mmHg, (*p* = 0.6160), respectively.

The rate of target BP attainment at 8 weeks, defined as sitSBP < 140 and/or sitDBP < 90 mmHg (for those with ≥60 years, sitSBP < 150 and/or sitDBP < 90 mmHg) was 84.76% (89/105) in olme/amlo/rosu group, 47.06% (48/102) in olme/rosu, and 76.92% (40/52) in olme/amlo group. This rate was significantly higher in the olme/amlo/rosu group as compared to the olme/rosu group (*p* < 0.0001) but did not differ between olme/amlo/rosu vs. olme/amlo group (*p* = 0.2272).

The rate of target LDL-C attainment according to the risk category of the NCEP ATP III guideline (for example, if risk factor 0–1, responder had LDL-C < 160 mg/dL, if risk factor ≥ 2 and 10 year risk ≤ 20%, responder had LDL-C < 130 mg/dL, and if coronary heart disease (CHD) or CHD risk equivalents or 10 year risk > 20%, responder had LDL-C < 100 mg/dL) was 84.76% (89/105) in olme/amlo/rosu, 83.33% (85/102) in olme/rosu and 15.38% (8/52) in olme/amlo groups, with no difference between 1st and 2nd groups, and significantly higher in 1st group as compared to 3rd group (*p* < 0.0001). Total cholesterol, TG and APO-B reduced significantly and HDL-C and APO-A1 increased significantly both in olme/amlo/rosu group and olme/rosu groups. However no change was found in olme/amlo group (Supplementary Table S4).

### 3.2. Subgroup Analysis

We stratified the patients according to age (65 years old over or not), sex and chronic kidney disease. Same findings were detected in all subgroups, more reduced sitSBP in olme/amlo/rosu group as compared to olme/rosu group and more lowered LDL-C in olme/amlo/rosu group as compared to olme/amlo group (Supplementary Table S5).

### 3.3. Safety Outcomes 

Total adverse drug events rate was 7.1% (19/265) in all population. 10.48% (11/105), and 5.66% (6/106) and 3.7% (2/54) in the olme/amlo/rosu, olme/rosu and olme/amlo groups, respectively; no serious adverse drug event occurred in all groups. No significant differences among the three groups were detected (*p* = 0.2163). Regarding the severity, mild drug reaction was most common (Table 4).

## 4. Discussion

Our study demonstrated the efficacy of the triple combination of olm/amlo/rosu on BP and LDL-C lowering, as well as safety, compared to olme/rosu or olme/amlo dual combination. Treatment with olme/amlo/rosu significantly reduced SBP by 24 mmHg and LDL-C by 52% compared to olme/rosu or olme/amlo. The attainment rates of target BP and LDL-C at 8 weeks were both 85%. Our data confirmed the efficacy of the triple combination of antihypertensive and anti-dyslipidemia drugs. In the safety profile, the olme/amlo/rosu combination shows similar results with the other two groups.

As the co-morbid and elderly population is growing more and more, the need for medication tends to increase. As such, drug compliance is likely to getting poorer as the number of pills increases. Recent studies have repeatedly provided evidence of the relationship of poor compliance and poor control of BP and LDL-C [18,19]. Poor compliance could be associated finally with poor clinical outcomes [20]. Therefore recent guidelines on hypertension management suggested single pill combination drugs to enhance drug compliance [12,21].

Our study had value in reflecting current the prevailing metabolic syndrome and testing the potential of a combination of different classes of drug, anti-hypertension and anti-dyslipidemia. These two diseases are frequently encountered in daily practice and are major components of metabolic syndrome. The drugs used in our study for BP lowering were olmesartan and amlodipine. Amlodipine is widely used in Korea as well as globally with myriad clinical data on improving clinical outcomes, as well as BP lowering, from large scale randomized clinical trials [17,21].

On the other hand, olmesartan is controversial regarding cardiovascular safety, because a ROADMAP trial showed more frequent development of fatal cardiovascular event in olmesartan users, 0.7% (15/2232) vs. 0.1% (3/2215), despite it improved primary endpoint of microalbuminuria. However, in that study, cardiovascular event was the secondary endpoint and the events rate was very small, 0.7% (15/2232) vs. 0.1% (3/2215) [14]. Moreover, the event was attributed to cardiovascular death among patients with preexisting coronary heart disease (2% [11/564] vs. 0.2% [1/540]). Considering our study population had simple hypertension and dyslipidemia (Table 1, only two patients have coronary heart disease) and the target patient group for this triple combination treatment was those with combined risks of simple hypertension and dyslipidemia rather than those with established coronary artery disease, olmesartan can be a good option for controlling hypertension. Other clinical studies performed after the ROADMAP trial and retrospective studies argue that the harm caused by olmesartan was not so robust and reported data for better cardiovascular outcome with that drug [15,16,22]. Noticeably, in a recent trial comparing BP target in elderly patients, one of the study drugs was olmesartan and this study showed reduced cardiovascular events in olmesartan users, so the previous concern can be diminished [16]. This drug also has potential for reducing albuminuria, improving renal function, improving left ventricular hypertrophy and halting coronary plaque progression [14,23,24].

The statin used in our study was rosuvastatin, and this also is associated with numerous data on improving hard clinical endpoints, especially in primary prevention of cardiovascular disease, as well as efficacy in lowering LDL-C [25,26]. Because rosuvastatin also has the property of delaying plaque regression, the combination of rosuvastatin with olmesartan could have potential in reducing or at least halting coronary plaque progression [27].

This combination of angiotensin receptor blockers (ARBs), angiotensin-converting enzyme inhibitors (ACEIs) and calcium channel blockers (CCBs) is first recommended as combination in major guidelines and is the most widely used combination in Korea and globally [17,21]. Thus there is a rationale for the two antihypertensive drugs with olmesartan and amlodipine in our trial. The combination of olmesartan/amlodipine was reported to be superior to perindopril/amlodipine in central BP reduction in a randomized, double blind trial [28]. When we consider that central BP lowering could play a role in improving outcomes, this result also supports the combination of olmesartan and amlodipine. In addition to this combination, we added rosuvastatin as a separate pill. This triple combination as SPC provided good safety and efficacy data and pharmacokinetic profile [29].

When we compare our results with other similar studies, the efficacy in attaining the BP target in our study was 85% which is equivalent to the olmesartan plus amlodipine plus hydrochlorothiazide triple combination of antihypertensive drugs, for which control rate was 83% from a large scale retrospective observational study [30]. In the safety profile, all adverse events occurred at 8.46% in that study, which was similar to our 7.17% [30].

The magnitude of reduction of LDL-C in olme/amlo/rosu arm (−52.41%) in our study, was very similar to that of the previous study (−52.3%) assessing the efficacy of olmesartan/rosuvastatin SPC reduction [11]. These results coincide well with previous studies, similar to ours in study design and drug used. Our results clearly demonstrated efficacy and safety and provide the rationale for developing a triple combination of olmesartan/amlodipine/rosuvastatin for treatment of hypertension and dyslipidemia.

Although this phase III trial was performed to develop the triple SPC drug for ease of drug compliance, the result is meaningful because it can provide physicians with efficacy and safety data for this SPC of antihypertensive and anti-dyslipidemia drugs and can help to treat patients with combined risks.

### Limitations

Our study has limitations. Firstly, a larger study population would be better to assess the primary end-point of sitSBP and LDL-C reduction and target level attainment after 8 weeks with three subset groups. Secondly, a larger population in olme/amlo could balance each group’s population and can give more concrete data, despite the ethical issue that the olme/amlo group do not receive anti-dyslipidemia drugs.

## 5. Conclusions

A triple combination of olmesartan/amlodipine/rosuvastatin treatment is safe and effective in reducing blood pressure and LDL-C. This combination will help to improve drug compliance in patients with co-morbidity. Future studies investigating whether this combination could increase the adherence rate and improve clinical outcomes are warranted.

## Figures and Tables

**Table 1:** Baseline characteristics.

	Olme/Amlo/Rosu (*n* = 105)	Olme/Rosu (*n* = 102)	Olme/Amlo (*n* = 52)	*p*-Value
Age (years), Mean (SD)	65.18 (9.34)	63.49 (9.76)	64.06 (8.94)	0.3817
Sex, *n* (%)				
Male	59 (56.19)	58 (56.86)	31 (59.62)	0.9176
Height (cm)				
Mean (SD)	162.64 (9.58)	161.31 (8.52)	162.21 (8.64)	0.5609
Weight (kg)				
Mean (SD)	70.96 (11.92)	69.48 (11.34)	70.50 (12.50)	0.6018
Body Mass Index (kg/m^2^)				
Mean (SD)	26.75 (3.28)	26.60 (3.01)	26.67 (3.26)	0.9949
Smoking Status, *n* (%)				0.7611
Never	57 (54.29)	56 (54.90)	30 (57.69)	
Current	21 (20.00)	26 (25.49)	10 (19.23)	
Former	27 (25.71)	20 (19.61)	12 (23.08)	
Drinking Status, *n* (%)				0.1590
Never	57 (54.29)	48 (47.06)	20 (38.46)	
Current	41 (39.05)	51 (50.00)	27 (51.92)	
Former	7 (6.67)	3 (2.94)	5 (9.62)	
Total Cholesterol (mg/dL)				0.6331
Mean (SD)	216.98 (34.82)	220.63 (35.24)	223.48 (37.24)	
HDL-C (mg/dL)				0.3811
Mean (SD)	49.23 (11.95)	46.87 (11.45)	48.65 (10.74)	
SBP (mmHg)				0.6207
Mean (SD)	153.58 (10.90)	153.71 (11.10)	151.30 (8.87)	
10-year risk assessment (score)				0.6538
Mean (SD)	16.38 (8.19)	17.22 (7.48)	16.25 (7.87)	
Risk Factor, *n* (%)				
hypertension	105 (100.00)	102 (100.00)	52 (100.00)	1
HDL-C < 40 mg/dL	25 (23.81)	24 (23.53)	13 (25.00)	0.7561
Age ≥45 in male, ≥55 in female	95 (90.48)	94 (92.16)	47 (90.38)	0.8543
Coronary heart disease	1 (0.95)	1 (0.95)	0	0.9978
Family history of premature CAD	8 (7.62)	9 (8.82)	4 (7.69)	0.3195

HDL, high density lipoprotein; CAD, coronary artery disease.

**Table 2:** Change of sitSBP at 8 weeks comparing olme/amlo/rosu and olme/rosu group.

SBP, mmHg	Olme/Amlo/Rosu (*n* = 105)	Olme/Rosu (*n* = 102)	*p*-Value
At Baseline, Mean(SD)	153.58 (10.90)	153.71 (11.10)	0.9639
At Week 8, Mean(SD)	129.28 (13.58)	144.00 (18.44)	<0.0001
Change form baseline at 8 week, Mean(SD)	−24.30 (12.62)	−9.72 (16.27)	<0.0001

**Table 3:** Change of LDL-C at 8 weeks comparing olme/amlo/rosu and olme/amlo group.

LDL-C, mg/dL	Olme/Amlo/Rosu (*n* = 105)	Olme/Rosu (*n* = 52)	*p*-Value
At Baseline, Mean(SD)	154.52 (30.84)	160.42 (32.05)	0.2672
At Week 8, Mean(SD)	72.72 (26.08)	153.81 (31.57)	<0.0001
Percent Change form baseline at 8 week, Mean(SD)%	−52.31 (16.63)	−2.98 (16.16)	<0.0001

**Table 4:** Adverse drug reactions.

Patients Number (%) (Event No)	Olme/Amlo/Rosu (*n* = 105)	Olme/Rosu) (*n* = 106)	Olme/Amlo (*n* = 54)	Total (*n* = 265)
Subjects with ADRs	11 (10.48)	6 (5.66)	2 (3.70)	19 (7.17)
95% Confidence Interval	(4.62, 16.33)	(1.26, 10.06)	(0.00, 8.74)	(4.06, 10.28)
*p*-value *				0.2163 (c)
Severity				
Mild	14	6	2	22
Moderate	0	2	0	2
Severe	0	0	0	0
Relationship with drugs				
Certain	0	0	0	0
Probable/Likely	0	2	1	3
Possible	7	3	0	10
Unlikely	7	3	1	11
Not related	0	0	0	0
Unassessable/Unclassifiable	0	0	0	0
Subjects with Serious ADRs	0	0	0	0
Exact 95% Confidence Interval	(0.00, 3.45)	(0.00, 3.42)	(0.00, 6.60)	(0.00, 1.38)
*p*-value *				NC
Subjects with ADRs Leading to drug Discontinuation	1 (0.95) (2)	0	0	1 (0.38) (2)
Exact 95% Confidence Interval	(0.02, 5.19)	(0.00, 3.42)	(0.00, 6.60)	(0.01, 2.08)
*p*-value *				0.6000 (f)
Subjects with ADRs Leading to Fatal circumstances	0	0	0	0
Exact 95% Confidence Interval	(0.00, 3.45)	(0.00, 3.42)	(0.00, 6.60)	(0.00, 1.38)
*p*-value *				NC

Olme, Olmesartan; Amlo, Amlodipine; Rosu, Rosuvastatin; ADR, adverse drug reaction; NC, not calculated. * Testing for difference among treatment groups, chi-square test (c) or Fisher’s exact test (f). Note: Denominator of percentage is the number of subjects in each group. Severity and relationship are displayed as ‘number of events’ and others are displayed as ‘number of subjects (percentage of subjects) (number of events)’. ADR is the adverse event whose relationship to the study drug is ‘Certain’, ‘Probable/Likely’, ‘Possible’, ‘Unlikely’, ‘Unassessable/Unclassifiable’.

## Data Availability

Not applicable.

## Supplementary Materials

The following supporting information can be downloaded at: https://www.mdpi.com/article/10.3390/jcm11020350/s1, Table S1. Inclusion criteria. Table S2. Pre-visit 2 inclusion criteria for dyslipidemia according to cardiovascular risk. Table S3. Final patients included. Table S4. Lipid levels other than LDL-C in 3 groups after 8week treatment. Table S5. Subgroup analysis.
