# Supplementary material for: A Prospective Randomized, Double-Blind, Multi-Center, Phase III Clinical Trial Evaluating the Efficacy and Safety of Olmesartan/Amlodipine plus Rosuvastatin Combination Treatment in Patients with Concomitant Hypertension and Dyslipidemia: A LEISURE Study
Source: J Clin Med. 2022 Jan 11;11(2):350. doi: 10.3390/jcm11020350 (PMC8779537; doi:10.3390/jcm11020350)
Supplement: Supplementary file 1 [file jcm-11-00350-s001.zip › jcm-1512875-supplementary.pdf]

Supplementary Tables

Supplementary Table S1. Inclusion criteria.

Classification of patients	Inclusion criteria	
	sitSBP (mmHg)	Lipid (mg/dL)
Patients taking only antihypertensive medications	< 180	100 ≤ LDL-C ≤ 250<br>Triglyceride < 400
Drug naïve Patients	≥ 140	
Patients taking only antidyslipidemic medications		LDL-C ≤ 250<br>Triglyceride < 400

Supplementary Table S2. Pre-visit 2 inclusion criteria for dyslipidemia according to cardiovascular risk.

Risk Factor Classification	Lipid (mg/dL)
Risk factor[1)] 0~1	160 ≤ LDL-C ≤ 250<br>Triglyceride < 400
Risk factor ≥ 2 and 10 year risk[2)] < 10%	
Risk factor ≥ 2 and 10% ≤ 10 year risk < 20%	130 ≤ LDL-C ≤ 250<br>Triglyceride < 400
CHD/CHD risk equivalents[3)]	100 ≤ LDL-C ≤ 250<br>Triglyceride < 400
10 year risk > 20 %	

1) Risk factor;
① Age(Male ≥ 45 years; Female ≥ 55 years, ② Family history of premature coronary artery disease (If any of the parents or siblings(Male < 55 years; Female < 65 years) has coronary artery disease), ③ Hypertension (BP≥140/90 mmHg or antihypertensive medication), ④Smoking, ⑤ Low HDL cholesterol level (HDL-C < 40mg/dL)
* High HDL cholesterol level(≥ 60mg/dL) is considered as a protective factor, and one factor is excluded from the total number of risk factors
2) Referring to Framingham Risk Score
3) Coronary artery disease or diabetes mellitus (HbA1c ≥ 6.5% or taking antidiabetic medications) or Other clinical forms of ASCVD (Peripheral artery disease, Abdominal aortic aneurysm, Carotid artery disease with symptom, etc.)

Supplementary Table S3. Final patients included.

		Treatment Group (Olme/Amlo/Rosu) (n=106)	Control Group 1 (Olme/Rosu) (n=106)	Control Group 2 (Olme/Amlo) (n=53)	Total (n=265)
Safety Set, n		105	106	54	265
Efficacy Set, n (%)	Full Analysis Set	105 (99.06)	102 (96.23)	52 (98.11)	259 (97.74)
	Per Protocol Set	94 (88.68)	88 (83.02)	42 (79.25)	224 (84.53)

*olme, olmesartan; rosu, rosuvastatin; amlo, amlodipine; v, visit

Supplementary Table S4. Lipid levels other than LDL-C in 3 groups after 8week treatment.

	**Olme/Amlo/Rosu**			**Olme/Rosu**			**Olme/Amlo**		
	**baseline**	**week 8**	△	**baseline**	**week 8**	△	**baseline**	**week 8**	△
Total cholesterol (mg/dL)	216.96	138.32	-78.64 (p<0.0001)	220.71	140.77	-79.93 (p<0.0001)	225.44	219.13	-6.31 (p=0.1165)
TG (mg/dL)	170.03	116.30	-53.72 (p<0.0001)	184.34	136.51	-47.83 (p<0.0001)	178.73	188.56	9.83 (p=0.8716)
HDL-C (mg/dL)	49.26	56.91	7.66 (p<0.0001)	46.91	52.95	6.04 (p<0.0001)	48.37	49.40	1.04 (p=0.3059)
APO-A_1 (mg/dL)	139.55	148.76	9.21 (p<0.0001)	138.16	147.47	9.30 (p<0.0001)	139.15	142.29	3.14 (p=0.1633)
APO B (mg/dL)	127.03	70.62	-56.41	130.86	76.02	-54.84	131.34	127.80	-3.54

			($p<0.0001$)			($p<0.0001$)			($p=0.2712$)

Supplementary Table S5. Subgroup analysis.

(A) SBP

**Age ≥65**	**Olme/Amlo/Rosu (N=61)**	**Olme/Rosu (N=51)**	P-value
At Baseline, Mean(SD)	153.75(10.98)	153.93(12.27)	0.8723
At Week 8, Mean(SD)	130.67(14.07)	147.89(19.44)	<0.0001
Change form baseline at 8week, Mean(SD)	-24.00	-9.00	<0.0001
**Age <65**	**Olme/Amlo/Rosu (N=61)**	**Olme/Rosu (N=51)**	P-value
At Baseline, Mean(SD)	153.34(10.92)	153.49(9.90)	0.7881
At Week 8, Mean(SD)	127.36(12.77)	140.10(16.69)	<0.0001
Change form baseline at 8week, Mean(SD)	-25.98(12.60)	-13.39(14.36)	<0.0001
**Male**	**Olme/Amlo/Rosu (N=59)**	**Olme/Rosu (N=58)**	P-value
At Baseline, Mean(SD)	153.65(10.97)	153.00(10.53)	0.7998
At Week 8, Mean(SD)	131.01(13.23)	139.61(15.71)	<0.0001
Change form baseline at 8week, Mean(SD)	-22.64(11.30)	-13.39(13.51)	<0.0001
**Female**	**Olme/Amlo/Rosu (N=46)**	**Olme/Rosu (N=44)**	P-value
At Baseline, Mean(SD)	153.50(10.93)	154.65(11.86)	0.7406
At Week 8, Mean(SD)	127.08(13.84)	149.78(20.29)	<0.0001
Change form baseline at 8week, Mean(SD)	-26.42(13.98)	-4.87(18.38)	0.0325
**Chronic kidney disease, Yes**	**Olme/Amlo/Rosu (N=7)**	**Olme/Rosu (N=4)**	P-value
At Baseline, Mean(SD)	150.91(10.83)	153.35(9.58)	0.5699
At Week 8, Mean(SD)	123.67(11.56)	152.10(13.21)	<0.0001
Change form baseline at 8week, Mean(SD)	-27.24(14.86)	-1.25(9.86)	0.0029
**Chronic kidney disease, No**	**Olme/Amlo/Rosu (N=98)**	**Olme/Rosu (N=98)**	P-value
At Baseline, Mean(SD)	153.77(10.94)	153.73(11.20)	0.9117

At Week 8, Mean(SD)	129.68(13.67)	143.66(18.60)	<0.0001
Change form baseline at 8week, Mean(SD)	-24.09(12.51)	-10.06(16.42)	<0.0001

(B) LDL-C

**Age ≥65**	**Olme/Amlo/Rosu (N=61)**	**Olme/Rosu (N=27)**	P-value
At Baseline, Mean(SD)	153.70(31.94)	156.63(20.83)	0.6111
At Week 8, Mean(SD)	71.72(24.22)	156.07(24.26)	<0.0001
Percent Change form baseline at 8week, Mean(SD) %	-52.43(15.41)	0.55(15.72)	<0.0001
**Age <65**	**Olme/Amlo/Rosu (N=44)**	**Olme/Rosu (N=25)**	P-value
At Baseline, Mean(SD)	155.66(29.58)	164.52(40.98)	0.3032
At Week 8, Mean(SD)	74.11(28.69)	151.36(38.32)	<0.0001
Percent Change form baseline at 8week, Mean(SD) %	-52.14(18.37)	-6.79(16.06)	<0.0001
**Male**	**Olme/Amlo/Rosu (N=59)**	**Olme/Rosu (N=31)**	P-value
At Baseline, Mean(SD)	150.63(29.97)	158.74(32.34)	0.2381
At Week 8, Mean(SD)	71.93(24.28)	148.68(31.07)	<0.0001
Percent Change form baseline at 8week, Mean(SD) %	-51.51(16.85)	-5.50(14.21)	<0.0001
**Female**	**Olme/Amlo/Rosu (N=7)**	**Olme/Rosu (N=4)**	P-value
At Baseline, Mean(SD)	159.52(31.54)	162.90(32.26)	0.6872
At Week 8, Mean(SD)	73.74(28.45)	161.38(31.52)	<0.0001
Percent Change form baseline at 8week, Mean(SD) %	-53.34(16.48)	0.75(18.41)	<0.0001
**Chronic kidney disease, Yes**	**Olme/Amlo/Rosu (N=7)**	**Olme/Rosu (N=4)**	P-value
At Baseline, Mean(SD)	161.14(27.67)	182.50(42.49)	0.9247
At Week 8, Mean(SD)	73.00(12.08)	140.50(39.82)	<0.0001

Change form baseline at 8week, Mean(SD)	-54.13(6.84)	-23.26(9.98)	0.0186
**Chronic kidney disease, No**	**Olme/Amlo/Rosu (N=98)**	**Olme/Rosu (N=48)**	P-value
At Baseline, Mean(SD)	154.05(31.13)	158.58(30.90)	0.4088
At Week 8, Mean(SD)	72.70(26.83)	154.92(31.05)	<0.0001
Change form baseline at 8week, Mean(SD)	-52.18(17.13)	-1.29(15.46)	<0.0001
